# A Magnetic Field Sensor Based on a Magnetic Fluid-Filled FP-FBG Structure

**DOI:** 10.3390/s16050620

**Published:** 2016-04-29

**Authors:** Ji Xia, Fuyin Wang, Hong Luo, Qi Wang, Shuidong Xiong

**Affiliations:** 1 Academy of Ocean Science and Engineering & College of Optoelectronic Science and Engineering, National University of Defense Technology, Changsha 410073, China; blovexiaji@163.com (J.X.); fuyin_wang@126.com (F.W.); luohongrong2000@163.com (H.L.); 2 College of Information Science and Engineering, Northeastern University, Shenyang 110819, China; wangqi@ise.neu.edu.cn

**Keywords:** magnetic field sensor, Fabry-Perot Cavity, magnetic fluid, Fiber Bragg Grating, temperature compensation

## Abstract

Based on the characteristic magnetic-controlled refractive index property, in this paper, a magnetic fluid is used as a sensitive medium to detect the magnetic field in the fiber optic Fabry-Perot (FP) cavity. The temperature compensation in fiber Fabry-Perot magnetic sensor is demonstrated and achieved. The refractive index of the magnetic fluid varies with the applied magnetic field and external temperature, and a cross-sensitivity effect of the temperature and magnetic field occurs in the Fabry-Perot magnetic sensor and the accuracy of magnetic field measurements is affected by the thermal effect. In order to overcome this problem, we propose a modified sensor structure. With a fiber Bragg grating (FBG) written in the insert fiber end of the Fabry-Perot cavity, the FBG acts as a temperature compensation unit for the magnetic field measurement and it provides an effective solution to the cross-sensitivity effect. The experimental results show that the sensitivity of magnetic field detection improves from 0.23 nm/mT to 0.53 nm/mT, and the magnetic field measurement resolution finally reaches 37.7 T. The temperature-compensated FP-FBG magnetic sensor has obvious advantages of small volume and high sensitivity, and it has a good prospect in applications in the power industry and national defense technology areas.

## 1. Introduction 

Magnetic field sensors have been widely applied for navigation, vehicle detection, current sensing, and spatial and geophysical researches. Compared with the other types of magnetic field sensor, all-fiber based sensors have been extensively investigated owing to their portability, high geometric adaptability, immunity from electromagnetic interference, long distance signal transmission for remote operation, and resistance to high pressure and corrosion.

Magnetic fluids (MFs) have numerous interesting optical characteristics [1,2,3], such as tunable refractive index, tunable transmittance, birefringence and thermal lens effects, *etc*. Generally, the components of a MF are magnetic particles, a carrier liquid and surfactants. When an external magnetic field or thermal effect is applied to a MF, then the refractive index of the MF varies with the changing magnetic field or thermal effect. Various MF-based optical devices have been developed [4,5,6], such as magnetic fluid light modulators, magnetic fluid optical gratings, and magnetic fluid optical fiber filters, *etc*.

Recently, various optical fiber structures with MFs have been proposed for magnetic field sensing, The employed structures include Fabry-Perot cavity [7,8], polymer optical fiber [9], fiber Bragg grating [10,11] multimode interference [12,13] Michelson interferometer [14] Sagnac interferometer [15,16] and photonic crystal fiber (PCF)-based interferometers [17,18,19]. Homa [7] used a MF-filled extrinsic FP interrogated with an infrared wavelength spectrometer to detect magnetic fields. The sensor readily measured the magnetic field with a range of 0.5 mT to 12.0 mT with corresponding sensitivities in the 0.3 to 2.3 nm/mT range. In 2014, Lv [20] proposed a novel fiber-optic magnetic field sensor, which was composed of an extrinsic fiber Fabry-Perot interferometer and a magnetic fluid. Preliminary experiments illustrated that the magnetic field measurement sensitivity was 0.0431 nm/Gs and the measurement resolution was better than 0.5 Gs in the range from 0 to 400 Gs. Their work however did not take the thermal effect of the MF into consideration. The refractive index of the MF behaves differently under varying magnetic field and temperature conditions, which causes a cross-sensitivity effect from the temperature and magnetic field existing in the MF. The measurement systems mentioned above don’t take the impact of the operating temperature on the characteristic refractive index into account, which makes the resulting magnetic field measurements unstable and inaccurate. In this work, a modified sensor probe based on the multiplex FP-FBG structure is put forward for temperature compensation, which solves the cross-sensitivity effect of the temperature and magnetic field in the MF. The FBG is written on the insert fiber end of the FP cavity, and it is sensitive to the temperature variation but insensitive to magnetic field changes. The resulting FP-FBG structure is experimentally demonstrated for high resolution magnetic field detection.

## 2. Principles of the FP-FBG Sensor Filled with Magnetic Fluid

**Figure 1:**
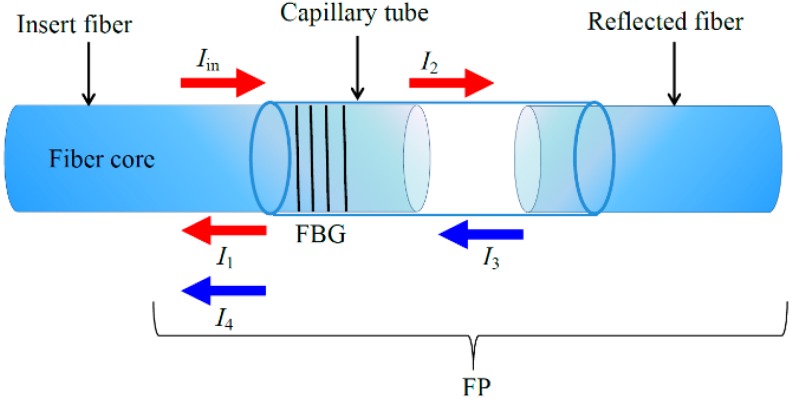
The structure of a FP-FBG sensor.

The multiplexing structure based on the FP-FBG sensor is shown in Figure 1, where the FBG is written on the insert fiber end of the FP cavity. When a broadband light beam with light intensity Iin transmits through the FBG, the reflected light intensity near the Bragg reflection wavelength is I1. Then the transmission light beam intensity I2 of FBG reaches the FP cavity. In a low-reflectivity FP cavity, the interference spectrum intensity of FP I_3_ is generated and transmitted back through the FBG once again. Finally, the reflected light beam intensity I*_out_* from the FP-FBG sensor is combined with the second FBG transmission light beam intensity I_4_ and the former FBG reflected light beam intensity I_1_, and the output light intensity of the FP-FBG sensor I*_out_* can be obtained as below:

(1) $$I_{out}=I_{1}+I_{4}=I_{in}\cdot [f_{FBG}+{\left(1-f_{FBG}\right)}^{2}\cdot f_{F-P}]$$

where, *f_FBG_* is the reflection coefficient of FBG and it is defined as $f_{FBG}=R\cdot \exp[-{\left(\lambda -\lambda_{B}\right)}^{2}/c^{2}]$, *R,,c* are the Bragg peak reflectivity, the center-reflected wavelength of FBG, and the bandwidth of the FBG reflected peaks. Accordingly, $f_{F-P}=2r\cdot [1+\cos(4\pi L/\lambda +\pi )]$ is the reflection coefficient of FP cavity, and *r* is the reflectivity of fiber end, *L* is the length of FP cavity.

**Figure 2:**
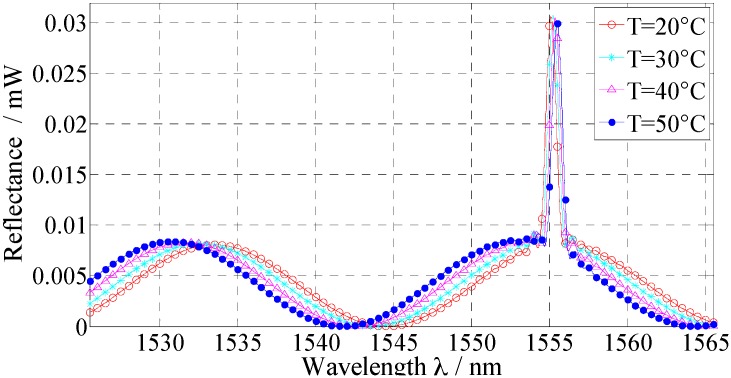
Simulation of the output spectrum under different temperatures (H = 0).

Meanwhile, the resonant peak wavelengths of FBG and FP should be set within a proper measurement wavelength-range from the input light and both of them can be distinguished through one another. In order to maintain the FP interference fringes and the FBG reflected peaks at a same order of magnitude, the reflectivity of FBG should be set to close to that of the low fitness FP sensor. Figure 2 shows the reflected spectrum simulation of the FP-FBG sensor.

In the simulation, the length of FP cavity is set as *L* = 40 μm, the center-reflected wavelength of FBG at room temperature 25 °C is $\lambda_{B}=1550\;\text{nm}$ with a reflectivity of 4%. The intensity of the incident light is 1 mW with a spectrum range of 1525 nm–1565 nm. The simulated spectrum of the FP-FBG is displayed clearly with combination of two reflected spectra at a same spectral resolution and the reflected spectra moves through one another without interference. As the temperature increases from 20 °C to 50 °C, the interference spectrum of the FP sensor shifts towards the short wavelength direction, however, the center-reflecting wavelength of the FBG moves towards along the long wavelength direction. In Figure 2, the amount of movement of the FBG resonant peaks is less than that of the FP interference fringes.

With reference to the research results of Chen *et al.* [21], under a constant temperature value of *T*, the relationship between the refractive index of the MF and the magnetic field is as below:

(2) $$n_{MF}=(n_{s}-n_{0})[\coth(\alpha \frac{H-Hc}{T})-\frac{1}{\alpha (H-H_{c})}]+n_{0}$$

where, *H*c is the critical value of the applied magnetic field (H>Hc), *n*_0_ is the refractive index of the MF with the critical magnetic field and *n*_s_ is the saturated refractive index of MF, and *α* is a fitting coefficient. Generally, the parameters of *n*_s_, *n*_0_, *α*, *H*c for a certain kind of magnetic fluid film are regarded as constants. Therefore, at a certain experimental temperature *T*, there is only one refractive index for the MF *n*_MF_ for a certain magnetic field *H*. The refractive index of the MF *n*_MF_ is tested under different magnetic field and temperature conditions, and the experimental results are used for magnetic field detection with temperature compensation. 

In the sensor probe with a FP-FBG structure, the FBG is written on the insert fiber end of the FP cavity. Being inspired by the concept that the central wavelength shifts of the FBG are applied to detect the external varying temperature, the central wavelength shift of FBG can be defined by Equation (3):

(3) $$\Delta \lambda_{B}=\lambda_{B}\left(\beta_{Tn}+\beta_{Tl}\right)\Delta T$$

where, *β_Tn_*, *β_Tl_* are the thermal-optical effect coefficient of 8.0 × 10^−6^/°C and thermal expansion effect coefficient of 0.55 × 10^−6^/°C in the FBG. ΔT is the variation value of the operating temperature. It is noted that the central wavelength shift of FBG is not sensitive to the magnetic field applied on the FP-FBG sensor, which is used for the measurement of magnetic field with temperature compensation. Due to the cross-sensitivity effect of the temperature and magnetic field in MF, the characteristic magnetic fluid refractive index are defined as follows:

(4) $$\Delta n_{MF}=\alpha_{Hn}\cdot \Delta H+\alpha_{Tn}\cdot \Delta T$$

where Δ*n_MF_* is the change of the magnetic fluid refractive index, and *α_Hn_*, *α_Tn_* are the magnetic field sensitive coefficient and temperature sensitive coefficient of the magnetic fluid, respectively. Considering the thermal expansion effect of optical fiber, the central wavelength shift in the output spectrum of the FP-FBG magnetic sensor is given by Equation (5):

(5) $$\Delta \lambda_{m}=\lambda_{m}\left[\alpha_{Hn}\cdot \Delta H+\left(\alpha_{Tn}+\alpha_{Tl}\right)\cdot \Delta T\right]$$

where, $\Delta \lambda_{m}$ is the central wavelength shift of the FP-FBG magnetic sensor, and *α_Tl_* is the optical fiber thermal expansion effect coefficient. Combining Equations (3) and (5), a relationship matrix between the FBG output wavelength shifts and the two under-test parameters is obtained in Equation (6). Equation (7) achieves the measurement of the temperature and magnetic field through calculating the matrix in Equation (6):

(6) $$\left[\begin{array}{c} \Delta \lambda_{m}\\ \Delta \lambda_{B} \end{array}\right]=\left[\begin{array}{cc} \alpha_{Hn}\lambda_{m} & \left(\alpha_{Tn}+\alpha_{Tl}\right)\lambda_{m}\\ 0 & \left(\beta_{Tn}+\beta_{Tl}\right)\lambda_{B} \end{array}\right]\left[\begin{array}{c} \Delta H\\ \Delta T \end{array}\right]$$

(7) $$\left[\begin{array}{c} \Delta H\\ \Delta T \end{array}\right]={\left[\begin{array}{cc} \alpha_{Hn}\lambda_{m} & \left(\alpha_{Tn}+\alpha_{Tl}\right)\lambda_{m}\\ 0 & \left(\beta_{Tn}+\beta_{Tl}\right)\lambda_{B} \end{array}\right]}^{-1}\left[\begin{array}{c} \Delta \lambda_{m}\\ \Delta \lambda_{B} \end{array}\right]$$

Obviously, when the spectrum drifts and the temperature detected by the FBG are measured in an experimental system, then the change of the magnetic field Δ*H* can be obtained by the matrix in Equation (7). According to Equation (7), the MF-filled FP-FBG sensor can achieve the simultaneous measurement of the magnetic field and the temperature, and the temperature detected in real-time can be used for the compensation of the magnetic field measurement. Finally, the cross-sensitivity effect of the temperature and magnetic field in the magnetic fluid is eliminated. 

## 3. Experiment System and Analysis

**Figure 3:**
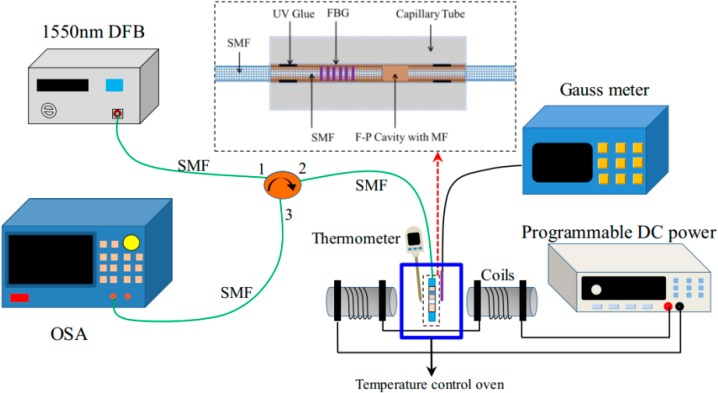
Experimental setup configuration for the FP-FBG sensing system. Inset: the cross-section schematic diagram of the FP sensing head.

The magnetic field measurement system setup is shown in Figure 3. The light beam output from a 1550 nm DFB is injected into the optical fiber circulator via port1, and it transmits into the MF-filled FP-FBG sensor for magnetic field and temperature detection. The programmable DC controls the power of the output electric current to generate a stable magnetic field through a set of coils. When the applied magnetic field varies, the refractive index of the MF changes and the light signal is modulated. The reflected light passes through port 2 to port 3 and it is detected by the spectrometer (AQ6370 OSA, YOKOGAWA, Tokyo, Japan, with a wavelength resolution of 0.02 nm). The MF-filled FP-FBG sensor is located in a temperature control oven (FLYSET Temperature Controller, Shenzhen Dragon Born Hot Runner Technology Co., LTD, Shenzhen, China, with a temperature resolution of ±1 °C) for the tests at different stable temperatures, and the thermometer is put close to the sensor head to detect the operating temperature. In addition, a Gauss meter is also located near the sensor head to measure the varying magnetic fields. The temperature variation detected by thermometer and the magnetic field variation detected by the Gauss meter are taken to compare with the measurement results of the MF-filled FP-FBG sensor. As shown in the inset of Figure 3, the MF-filled FP-FBG sensor has a FBG length of 10 mm with a refractive index of R = 4% (the grating wavelength is 1550 nm). The length of MF filled in the FP cavity is fabricated with 32 μm. First, the fiber end written with a FBG inserts into the capillary tube; after the fiber is aligned with the capillary it is observed under a microscope; the MF (EMG605, Ferrotec USA Corporation, Bedford, NH, USA) with a volume content of C = 1.8% is infiltrated into the capillary tube. Finally, the reflected fiber is inserted into the capillary tube and sealed with UV glue under ultraviolet light irradiation for 24 h.

**Figure 4:**
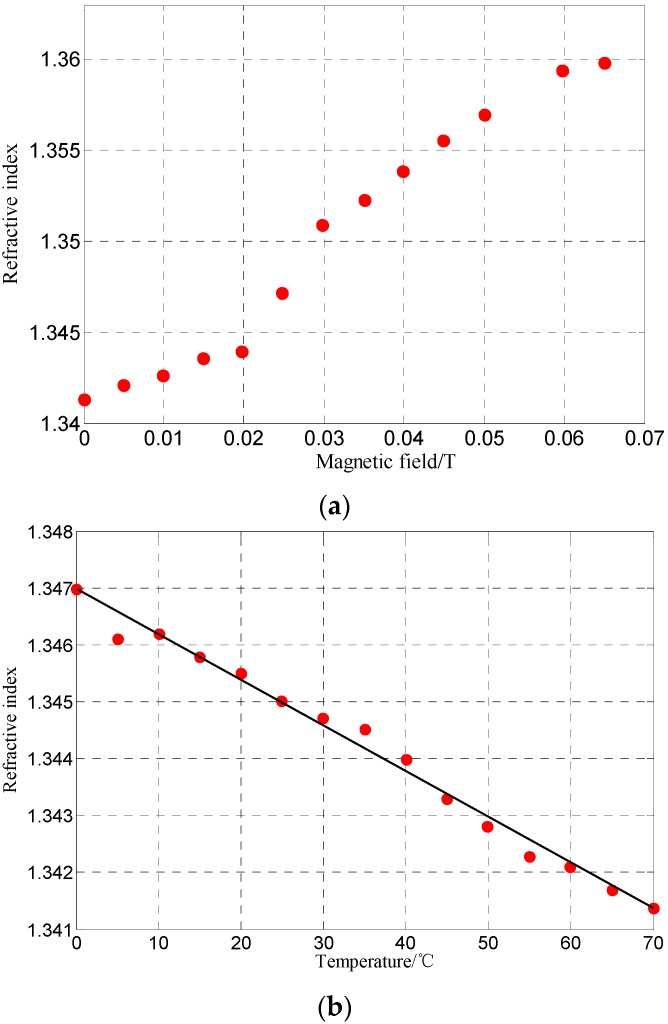
(**a**) Magnetic fluid refractive index variation *versus* transverse magnetic field; (**b**) Magnetic fluid refractive index variation *versus* temperature.

To illustrate the operation, the optical fiber end face reflection method based on the Fresnel reflection principle is performed to measure the characteristics of refractive index of the MF under different magnetic fields and temperatures, as shown in Figure 3. The refractive index of the MF under parallel magnetic fields behaves as shown in Figure 4a, which shows that *n*_MF_ = 1.3414 without any applied magnetic field.

**Figure 5:**
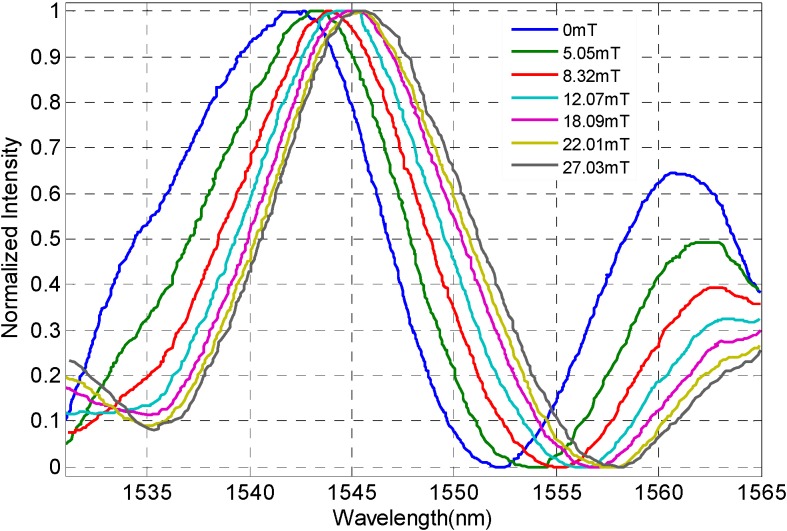
Spectrograms under a magnetic field range of 0~30 mT.

When *H* is less than 20 mT, *n*_MF_ increases gradually while the variation is very little. With the *H* increases from 20 mT to 60 mT, the corresponding *n*_MF_ increases from 1.3446 to 1.3600 with high sensitivity, which plays an important role in determining the effective operating magnetic field range in the experiment. Although *H* continues to increase from 60 mT, *n*_MF_ does not change obviously, which indicates that *n*_MF_ has reached its saturation value under the strong magnetic field. Therefore, the MF has an effective working magnetic field range in the experiment, and the range of magnetic field *H* is set from 0 mT to 40 mT. When the direction of magnetic field is transverse to the direction of the incident light, and the polarizability of MF increases with the increase of the magnetic field (magneto-electric effect). The change of *n*_MF_ is similar to the performance of MF polarizability, and it is dependent on the counter-direction between the applied magnetic field and the light transmitting through the MF. When the magnetic field is set as 0 T, the refractive index of MF is shown in Figure 4(b). As the temperature varies from 0 °C to 70 °C, the refractive index of the MF decreases linearly with a temperature sensitivity of −0.00008 RIU/°C. When MF is simultaneously subjected to an operating magnetic field and a changing temperature, the refractive index of the MF behaves according to both of them, making it hard to determine the refractive index of the MF as it responds to the the changing magnetic field or changing temperature and this will lead to an inaccurate magnetic field detection. Hence, it is necessary to take effective measures to compensate the operating temperature in the magnetic field measurement. With PW*@work the temperature in the experiment system is set to 25 °C, and the FP-FBG sensor filled with MF is located in a temperature control oven between a set of coils. The magnetic field direction is transverse to that of the incident light. As the applied magnetic field controlled by the programmable DC power increases from 0 mT to 39.12 mT, the normalized interference spectrum of the FP-FBG sensor shift towards the long wavelength direction (near the wavelength of 1550 nm) as shown in Figure 5. 

**Figure 6:**
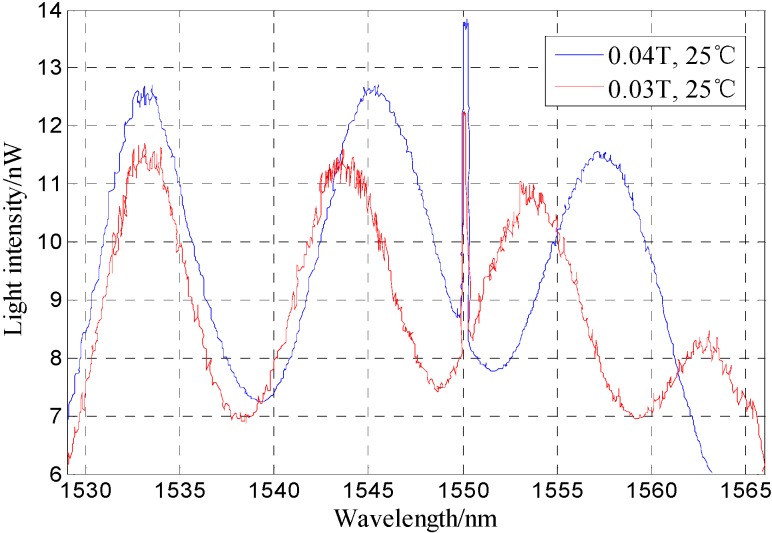
Spectrograms under a magnetic field range of 0~30 mT.

Figure 6 gives the FP-FBG sensor test results under two magnetic fields. At room temperature (25 °C) it can be obtained that the interference fringes of FP move with the magnetic field variation but the reflected peak of the FBG is unchanged.

**Figure 7:**
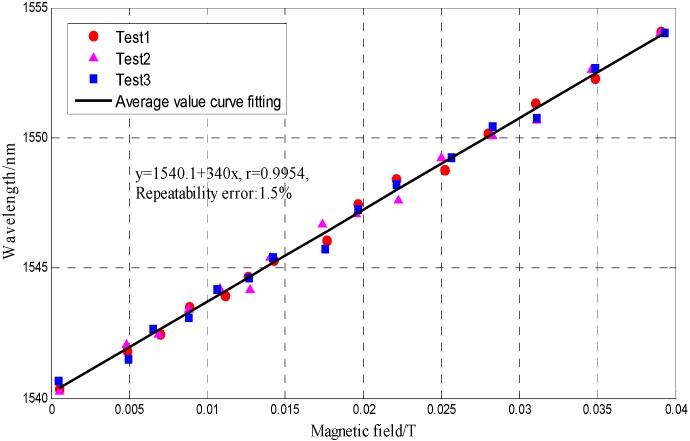
FP peak wavelength variation *versus* magnetic field.

The result in Figure 6 is in good agreement with the conclusion that the FBG written in the insert end of FP cavity is insensitive to magnetic field changes. The tests (the test was repeated three times) for a MF-based FP-FBG sensor under different magnetic fields are shown in Figure 7, where the curve fitting of the relationship between resonance peak drifts and the magnetic field indicates the MF-based FP-FBG sensor has a magnetic field sensitivity of 0.34 nm/mT with a repeatability error of *R*^2^ = 1.5%.

**Figure 8:**
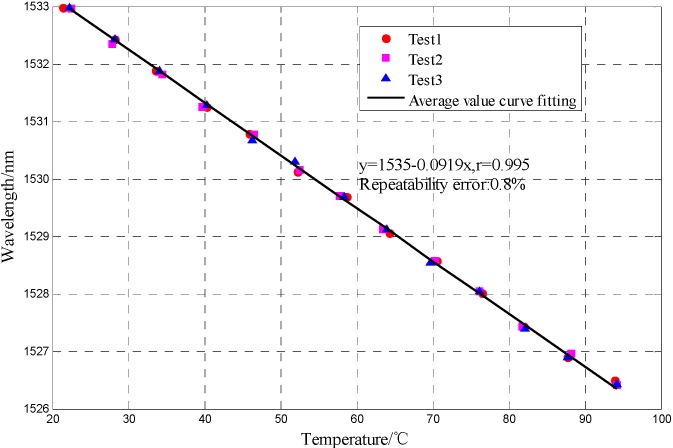
FP peak wavelength shift variation *versus* temperature.

**Figure 9:**
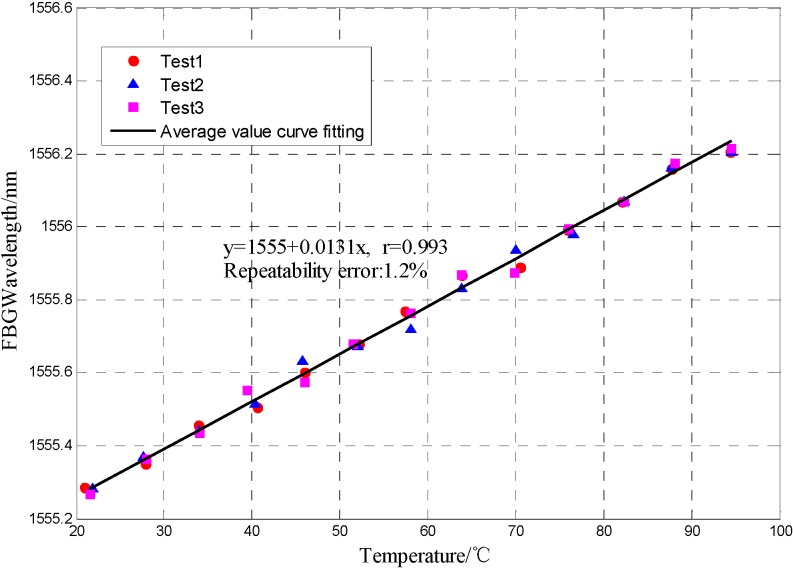
FBG wavelength shift variation *versus* temperature.

The interference spectrum of the MF-filled FP-FBG sensor which shifts under different temperature behaves as seen in Figure 8 and Figure 9.

Considering that the output spectrum of the MF-filled FP-FBG sensor is combined with the reflected spectra of the FP sensor and FBG sensor and their resonance peak shifts, the analysis of the interference spectrum of FP-FBG sensor under different temperatures can be equally separated into those of the FP sensor and FBG sensor. As the temperature increases, the refractive index of the MF decreases and the output interference spectrum of the FP sensor shifts in a shorter wavelength direction. When the resonance peak of the FP sensor moves within the temperature range 20 °C < T < 95 °C, the MF-filled FP-FBG sensor has a temperature sensitivity of 0.092 nm/°C with a repeatability error *R*^2^ = 0.8%. As illustrated in Figure 9, the center wavelength of the FBG sensor shifts less than that of the FP sensor, and the FBG has a temperature sensitivity of 0.013 nm/°C with a repeatability error *R*^2^ = 1.2%.

Finally, when the magnetic field and temperature are applied to the MF-filled FP-FBG sensor, the corresponding magnetic field and temperature can be calculated from Equation (7) combined with the analysis of Figure 7, Figure 8 and Figure 9. In the system, the temperature measurement can be used as a compensation for the magnetic field detection by the FP-FBG sensor filled with MF. According to Equation (7), a magnetic field sensitivity of 0.34 nm/mT, temperature sensitivity of 0.092 nm/°C in the MF-filled FP sensor and a temperature sensitivity of 0.013 nm/°C in the FBG sensor can be obtained:

(8) $$\left[\begin{array}{c} \Delta H\\ \Delta T \end{array}\right]={\left[\begin{array}{cc} 0.34\;\text{nm}/\text{mT} & -0.092\;\text{nm}/℃\\ 0 & 0.013\;\text{nm}/℃ \end{array}\right]}^{-1}\left[\begin{array}{c} \Delta \lambda_{m}\\ \Delta \lambda_{B} \end{array}\right]$$

**Figure 10:**
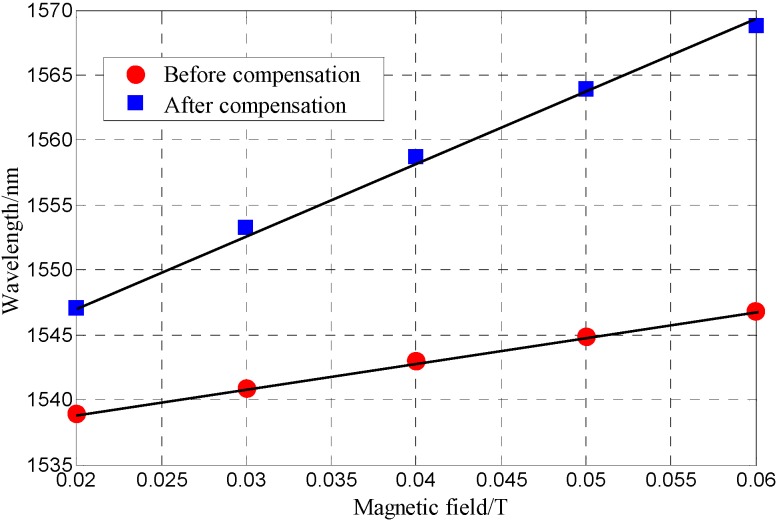
The test comparison of magnetic field sensitivity in the MF-filled FP-FBG sensor.

Based on the sensing characteristic matrix in Equation (8) of the FP-FBG magnetic sensor, the MF-filled FP-FBG sensor (after compensation) and the MF-filled FP sensor (before compensation) are simultaneously tested in the magnetic field range 0.02T < H < 0.06T. First, the temperature in the sensor head area is accurately measured by the reflected peak wavelength of the FBG; second, the wavelength of the FP-FBG varying with the magnetic field is modified according to the sensing characteristic matrix in Equation (8) and the magnetic field with temperature compensation is obtained in Figure 10. Figure 10 illustrates the fitted curves based on the test points. The magnetic field sensitivity after compensation is improved to 0.53 nm/mT with the comparison of the magnetic field sensitivity before compensation of 0.23 nm/mT. As the wavelength measurement resolution of OSA is 20 pm, the magnetic field measurement resolution of FP-FBG sensor could reach 37.7 μT.

## 4. Conclusions

Based on the characteristics of the refractive index of a MF, a fiber optic FP-FBG magnetic field sensor is proposed and demonstrated for magnetic field measurements with temperature compensation. The key point in the magnetic field measurement is to overcome the cross-sensitivity effect of the temperature and magnetic field in the MF. The FBG is written on the insert fiber of FP cavity for the operating temperature detection. Due to the fact the FBG is sensitive to temperature variations, the operating temperature in the sensing area can be measured and compensated for the magnetic field measurement. Preliminary experimental results show that the sensitivity of magnetic field measurement could reach 0.53 nm/mT and the magnetic field measurement resolution could reach 37.7 µT. The FP-FBG magnetic field sensor probe has the advantages of simple structure, easy fabrication, anti-corrosion properties, low cost, and so on, and this sensor would find potential applications in the measurement of electromagnetic fields.
